# Isolation, differentiation, and characterization of mesenchymal stem cells from human bone marrow 

**Published:** 2017

**Authors:** Kaveh Baghaei, Seyed Mohmoud Hashemi, Samaneh Tokhanbigli, Ali Asadi Rad, Hamid Assadzadeh-Aghdaei, Abdolhamid Sharifian, Mohammad Reza Zali

**Affiliations:** 1 * Basic and Molecular Epidemiology of Gastrointestinal Disorders Research Center, Research institute for Gastroenterology and Liver Diseases, Shahid Beheshti University of Medical Sciences, Tehran, Iran.*; 2 * Department of Immunology, School of Medicine, Shahid Beheshti University of Medical Sciences, Tehran, Iran *; 3 *Erfan Niayesh Grand Hospital, Tehran, Iran *; 4 * Gastroenterology and Liver Diseases Research center, Research institute for Gastroenterology and Liver Diseases, Shahid Beheshti University of Medical Sciences, Tehran, Iran.*

**Keywords:** Mesenchymal Stromal Cell, Flow cytometry, Differentiation

## Abstract

**Aim:**

We describe the minimum requirements and a simplified method for isolation and characterization of mesenchymal stem cells (MSCs) from human bone marrow.

**Background:**

MSCs are well known adult stem cells present in many tissues such as adipocytes, chondrocytes, osteoblasts, and neurons. Many isolations and characterization methods have emerged to apply MSCs in the clinical applications, which many of them are expensive and time-consuming.

**Methods:**

MSC isolation was carried out from human bone marrow, and cultured in defined medium. Cultures were maintained at 370C in a humidified atmosphere containing 5% CO2 for 48h. The medium was exchanged every 3-4 days. Adherent cells were characterized according to main criteria defined by ISCT, such as differentiation capability to adipocyte and osteoblast using specific differentiation mediums; also, flow cytometry verified MSC specific markers.

**Results:**

Isolated MSCs had a fibroblastic-like appearance with adherent property to the culture plate. Differentiation function was proved with the formation of lipid drops and calcium oxalates on the differentiated MSCs and finally, purified MSCs from bone marrow were positive for cell surface markers, CD73, CD90, and CD105 while being negative for CD34 and CD45.

**Conclusion:**

These findings confirm that the represented method is capable of isolating MSCs from bone marrow with proven results according to all minimum criteria defined by the International Society for Cellular Therapy (ISCT).

## Introduction

 Mesenchymal stem cells (MSCs) are known as multipotential stem cells with features of self-generation and differentiation to a variety of cell types such as adipocytes, chondrocytes, osteoblasts, and neurons (1). MSCs are easily isolated from different sources like adipose tissue, tendon, peripheral blood, and cord blood (2–4). They are expandable, immunosuppressive and also do not stimulate immediate immune responses. Therefore, MSCs are a popular candidate in regenerative medicine (5–7).

As an emerging science, regenerative medicine applies the living cells and tissues to treat diseases incurable by conventional therapies. Cell-based therapies, as an important aspect of this field, are used in the treatment of cancers and autoimmune diseases. Human mesenchymal stem cells (hMSCs) are introduced as a leading candidate in regenerative medicine and cell therapy era (8).

Cellular therapies using MSCs are based on either unselected autologous Bone marrow (BM) cells or specific sub-populations, which especially require MSC isolation. BM is the most common source of MSCs. MSCs have been successfully isolated and characterized from many species including pig, rat, rabbit, dog, sheep, pig, mouse and human (9–13).

Hematopoietic stem cells and BM-MSCs are two main stem cell populations which can be isolated from BM (14). The most important property of BM-MSCs, which is used in isolation and purification process, is their physical adherence to the plastic cell culture plate (15). A variety of techniques have been used for isolation and enrichment of MSCs, including antibody-based cell sorting (16), low and high-density culture techniques (17, 18), positive negative selection method (19), frequent medium changes (20), and enzymatic digestion approach (21). 

Regardless of the isolation method, isolated MSC should fulfill certain criteria. A gold standard method for isolation of MSC has been described by Pittenger et al (22). Mesenchymal and Tissue Stem Cell Committee of the International Society for Cellular Therapy (ISCT) proposed minimal criteria to define human MSCs. First, MSCs are plastic-adherent cells when they are maintained in standard culture conditions. Second, MSCs should express CD105, CD73, CD90, and lack expression of CD45, CD34, CD14, CD11b, CD79 alpha or CD19 and HLA-DR surface molecules. Third, MSCs should be able to differentiate to osteoblast(s) and adipocyte(s) in vitro. 

Plastic adherence is one of the well-known properties of MSC, and unique subsets of MSCs exert this feature as described before (23). Surface Ag expression is one of the advantages which allows for rapid identification of a cell population by flow cytometery or other similar techniques. To characterize MSCs, it has been shown that these cells should express CD105, CD73, and CD90. To distinguish MSCs from hematopoietic stem cells, it is recommended that lack of expression of hematopoietic Ag should be checked as additional criteria for MSCs. In this regard, it is suggested that a panel of antibodies should be used to exclude the most likely cells in MSC cultures. CD45 is a leukocyte marker, CD34 is expressed on hematopoietic progenitors and endothelial cells, CD14 and CD11b are known markers for monocytes and macrophages, CD79a and CD19 are markers of B cells and HLA-DR molecules are not expressed on MSCs unless stimulated by different factors such as IFN-gamma. It is possible that each laboratory tries with a special panel of antibodies to recognize which are most reliable. In addition to Ag recognition, another way to confirm the characterization of MSCs is their capacity for differentiation. The capacity of MSCs to differentiate into osteoblasts and adipocytes is used as a standard in vitro to distinguish the MSCs. Differentiation to osteoblasts could be demonstrated by staining with Alizarin Red or von Kossa staining. Adipocyte differentiation is readily demonstrated by staining with Oil Red O. In our study, we present the minimum requirements and a simplified method for isolation and characterization of MSCs from human bone marrow. 

## Methods

**Bone Marrow (BM) Extraction**

Human bone marrow was obtained from the iliac crest of patients treated for transplantation in the Taleghani Hospital after having obtained their written consent. BM was collected aseptically into K2EDTA tube. The buffy coat was isolated by centrifugation (450 × g, 10 min), suspended in 1.5 mL PBS, and used for culture. The separated buffy coat was layered onto equal volume of Ficoll (GE health care, USA) and centrifuged (400 × g, 20 min). Cells at the interface were removed, and washed twice in sterile PBS.

**Mesenchymal stem cell culture**

Human bone marrow progenitor cells were cultured on tissue treated culture plates in DMEM medium supplemented with 10% FBS and penicillin/streptomycin (50 U/mL and 50 mg/mL, Gibco-Invitrogen, Carlsbad, USA; respectively). The plates were maintained at 37°C in a humidified atmosphere containing 5% CO2 for 48 h. To exchange the medium, the plates were washed with PBS in order to remove non-adhered cells and the medium was replaced. The cultures were maintained for an additional week with one medium exchange. 

**Characterization of human mesenchymal stem cells by differentiation**

To characterize the adherent cells, osteoblastic differentiation was induced by culturing confluent human MSCs for 3 weeks in osteoblastic differentiation media (all from Sigma) and after three weeks, the cells were stained by Alizarin. To induce adipocyte differentiation, confluent MSCs were cultured 1 to 3 weeks in differentiation medium, and lipid droplet staining was carried out by S Red Oil (Sigma).

**Flow cytometric analysis**

Flow cytometry was used to assess the immune profile of MSCs, using the standard for MSC as described by the International Society for Cellular Therapy (ISCT) (23) Cells (P2-3) were harvested, pelleted and resuspended in 1% bovine serum albumin (BSA in PBS), and counted. Each population containing 105 cells was used for flow cytometry. Cells were stained with directly PE (phycoerythrin) conjugated antibodies against CD14, CD34, CD45, CD90, CD105 and CD73 (ebioscience, Germany). An appropriate isotype-matched control antibody named mouse IgG1 K Iso control (ebioscience, Germany) was used in all analyses. Cells were analyzed on FACS flow cytometry using Cell Quest Software (Becton Dickinson, UK). 

## Results

**Human MSC isolation and culture**

**Figure 1:**
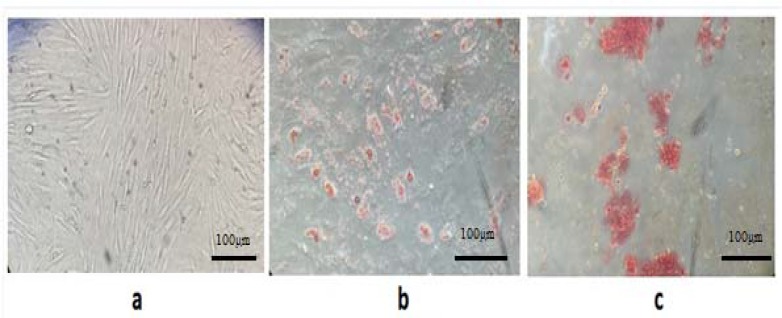
**Microscopic images of human mesenchymal stem cells isolated from bone marrow. (a) hMSCs spindle-shaped fibroblast- like appearance, extended in opposite directions from a small cell body (20X) (passage number 2). (b) adipogenic-induced hMSC, intracellular staining using oil red-O. (c) Osteogenic differentiation assay, Alizarin staining specifically shows calcium oxalates in differentiated hMSCs (5 days in differentiation medium, passage number 2-3**

Human MSC Isolation was achieved by Ficoll-Paque gradient centrifugation. The MSC like population formed a single layer, which allowed for more effective MSC extraction (24). The harvested cells were cultured in tissue treated culture dishes; thus, only hMSCs adhered and were maintained in media. After 8–12 days, the majority of non-adherent cells were removed during the medium exchanges. The remaining cells had a heterogeneous fibroblastic-like appearance and exhibited distinct colony formation (Fig1a). The hMSC cultures showed increased proliferation, which gradually resulted in maintaining a homogeneous fibroblastic morphology. hMSCs have mainly a spindle-shaped appearance with extension in opposite directions from a small cell body (Fig1a). 

**Figure 2:**
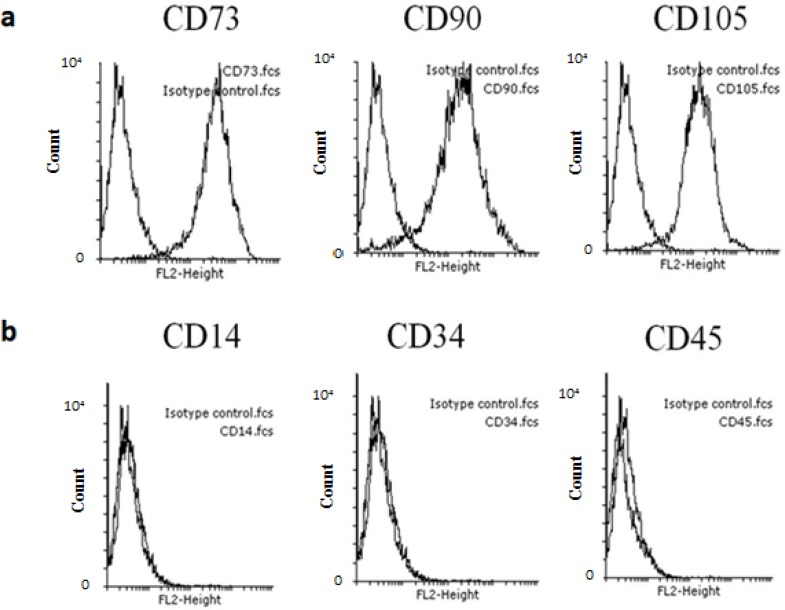
Flow cytometry analysis of cell surface markers present on hMSCs derived bone marrow. (a) positive markers CD73, CD90, and CD105 (b) while being negative for CD14, CD34and CD45

**Functional characterization**

hMSCs are defined as multipotent stem cells that should be able to differentiate to specific lineages like osteoblastic and adipocytic (22). Therefore, to determine whether isolated MSCs were capable of differentiate toward these two lineages, the osteogenic differentiation assay was performed. Alizarin staining clearly showed the formation of calcium oxalates on the differentiated MSCs, which was not observed in the undifferentiated cells (Fig 1c). Intracellular lipid droplets staining using oil red- O proved the adipogenesis of hMSCs (Fig 1b). These observations were absent in the undifferentiated hMSCs. These findings confirmed the characterization of cells as hMSCs and show the potential of MSC to differentiate to these lineages, i.e. osteogenic, and adipogenic.

**hMSC surface markers expression**

A minimal immune positive criterion for the identification of MSCs cells is the presence of CD73, CD90, and CD105 while being negative for CD34, CD45. Purified MSCs from bone marrow and adipose could be easily characterized by cell markers expressed on their surface. Based on available Abs for hMSC, this study elucidated that hMSCs were positive for CD73, CD90, and CD105 (Fig 2a), but were negative for CD34, CD45, and CD14 (Fig. 2b). 

## Discussion

After ethical concerns about embryonic stem cells (ESCs), much attention has been drawn towards adult stem cells, especially mesenchymal stromal cells (MSCs). These cells are considered as multipotent which are able to differentiate into a variety of cells such as adipocytes, chondrocytes, osteoblasts, and neurons. One of greatest aspects of these cells is the immunomodulatory feature, which makes them a preferable candidate in regenerative medicine. Many isolation and characterization methods have emerged to apply MSCs in the clinical application, many of which are expensive and time-consuming. 

This study presented a simplified technique to isolate hMSCs from bone marrow. Identification of hMSCs was carried out based on the following findings: (i) the hMSCs were adherent to plastic and exhibited fibroblastic spindle shapes with extension in opposite directions from the cell body as observed under phase contrast microscope. (ii) The specific antigen expression in hMSCs was identified by flow cytometry, i.e. CD14, CD34, CD45, CD90, CD105 and CD73; and (iii) histology staining (Alizarin, and oil-red-O staining) indicated that hMSCs were capable of lineage differentiation in appropriate medium for osteogenic and adipogenic differentiation. 

Experiments have demonstrated that isolated hMSCs between P2- P3 will produce sufficient cells for analyses. According to the ISCT guidelines (23), one of the characterization criteria for hMSC is phenotypic co-expression of CD105, CD90 and CD73 (> 95%), and lack of CD34, CD45, CD14 (< 2%). Flow cytometry results illustrated that the assessed hMSC populations consistently fulfilled all hMSC criteria. A previous study has proved that bone marrow-derived MSCs in culture have a heterogeneous CD34 and CD45 phenotype that changes under in vitro conditions (25). In this study, qualitative assays were used to determine the in vitro multi-lineage developmental potential of hMSCs after in vitro exposure to specific culture conditions. The hMSCs used in this study had a propensity to differentiate into the osteogenic and adipogenic lineages based on the qualitative (histological staining) results. 

With the results of the present study, hMSCs can be isolated by the established method. All the criteria, such as the plastic adherent behavior, the described morphological appearance, expression of selected CD markers, and the ability to undergo lineage differentiation, were fulfilled by these cells.
